# Diagnostic Capability of Isolated-Check Visual Evoked Potential for Early to Moderate Primary Open-Angle Glaucoma

**DOI:** 10.3390/life13061257

**Published:** 2023-05-25

**Authors:** Xia Wang, Yuan Fang, Ruoshi Li, Yingzi Pan

**Affiliations:** Department of Ophthalmology, Peking University First Hospital, Beijing 100034, China

**Keywords:** glaucoma, open angle, isolated-check, diagnosis, visual evoked potential

## Abstract

This study aimed to evaluate the diagnostic capability of isolated-check visual evoked potential (icVEP) for primary open-angle glaucoma (POAG) via comparison with visual field (VF) tests and pattern visual evoked potential (PVEP). This cross-sectional study enrolled 68 subjects, including 33 POAG patients and 35 controls. All subjects underwent a complete ophthalmic examination, including icVEP, PVEP, and VF tests. The diagnostic performance, the area under the receiver operating characteristic curve (AUC), the integrated discrimination index (IDI), and the net reclassification index (NRI) were calculated. The clinical benefits of the three tests were compared via decision curve analysis (DCA) of the signal-to-noise ratio (SNR) from icVEP, the P100 latency and amplitude of 1° and 0.25° checks from the PVEP, pattern standard deviation (PSD), and mean deviation (MD) from the VF test. The SNR, MD and PSD, PVEP P100 latency of 0.25° checks, and P100 amplitude (both 1° and 0.25° checks) showed significant differences between the POAG and control groups (* *p* < 0.05), except for the P100 latency of 1° checks. Regarding diagnostic ability, the three tests, AUC, IDI, and NRI, showed no significant difference (*p* > 0.05). The DCA showed that the clinical benefits of icVEP (SNR) were comparable to those of VF (MD and PSD) and better than those of PVEP (P100 latency and amplitude). In addition, no significant difference was found in the consistency analysis of the qualitative comparison between the icVEP, VF, and PVEP (McNemar *p* > 0.05). In this study, icVEP showed a diagnostic ability for early to moderate POAG patients comparable to VF and PVEP. IcVEP might be applied as a supplementary psychophysical examination method in addition to VF examinations for special POAG populations who have difficulty cooperating with the VF examination.

## 1. Introduction

Primary open-angle glaucoma (POAG) is one subtype of glaucoma and is the leading cause of irreversible blindness worldwide [1]. Standard automated perimetry (SAP) is the gold standard for glaucomatous visual function detection. However, the reliability of SAP results is largely dependent on the cognitive state of the patient [2]: the patient has to be able to understand how to perform the test, remain focused on the task, and be able to respond to every visual stimulus observed; therefore, SAP examination is difficult to perform for populations with poor hand–eye coordination, such as upper limb disability, and Alzheimer’s disease patients. Another functional impairment test is electrophysiology, which is relatively objective compared with SAP because the patient does not need to respond to visual stimuli during the test process. Visual evoked potentials (VEPs) are one kind of electrophysiological approach to assessing the function of the visual system. P100 latency and P100 amplitude assessed via pattern VEPs (PVEPs) have been reported to detect functional damage in glaucoma patients [3,4,5]. One of the advantages of VEP detection is that it only requires the gaze of the subject without human–machine conversations; thus, it does not require good understanding or hand–eye coordination.

However, traditional VEP protocols require patients to maintain a long fixation duration, the interpretation of results is often subjective, and an experienced physician is required [6]. Recently, isolated-check VEPs (icVEPs), a new visual electrophysiology technology, have been reported to be able to detect glaucomatous visual damage early and quickly [7]. In recent years, this technique has been applied to detect optic nerve damage of glaucoma [8,9,10,11,12,13] and other diseases such as traumatic optic neuropathy [14] and dysthyroid optic neuropathy [15,16]. The specificity and sensitivity values in detecting glaucomatous optic nerve defects are 84.6–100% and 53.1–83%, respectively [7,8,9,10,11].

Retinal ganglion cells (RGCs) mainly consist of parvocellular (P) cells, magnocellular (M) cells, and other types, which correspond to different visual pathways in the brain [17,18]. P cells account for approximately 80% of RGCs, while M cells account for only 10% [19,20]. Because the ratio of healthy M cells to total M cells in glaucoma patients approaches zero more quickly than the ratio of P cells, isolating M cells via specific stimulation may help in detecting glaucoma early [19,20]. IcVEPs can provide low spatial/high temporal frequency stimulations specific to the M cell pathway [7,8], thus providing a basis for detecting glaucomatous function damage.

The purpose of this study was to assess the diagnostic value of the icVEP method by comparing it with PVEP and visual field (VF) tests in POAG and control populations.

## 2. Materials and Methods

### 2.1. Participants and Criteria

This was a cross-sectional study that complied with the Helsinki Declaration. Informed consent was obtained from each subject.

All participants were recruited from the Department of Ophthalmology at Peking University First Hospital from November 2019 to November 2020. The inclusion criteria were as follows: ≥18 years old and spherical refraction error within −6 to +4 diopters (D) with reliable VFs. The criteria for POAG patients were the following: the presence of glaucomatous optic nerve damage (cup/disc ratio(C/D) > 0.6 or an asymmetry difference of C/D > 0.2; the presence of neuroretina rim thinning, excavation, notching, or characteristic retinal nerve fiber layer (RNFL) defects [21]), as judged based on stereoscopic fundus photography by two glaucoma specialists (PYZ, FY); and an open-angle in gonioscopy. Early- and moderate-stage POAG patients were included according to the Hodapp–Anderson–Parrish criteria [22]. Age-matched control subjects were recruited voluntarily, and those with an intraocular pressure (IOP) of ≤21 mmHg, C/D < 0.6, no disc damage, no RNFL defect in a stereoscopic fundus examination, and no family history of glaucoma were included. Disagreements between the two specialists were settled via negotiation. The exclusion criteria included congenital or secondary glaucoma; a history of ocular trauma and intraocular surgery (such as cataract surgery or vitrectomy); a best-corrected visual acuity (BCVA) of less than 20/40; poor fixation; retinal and optic nerve diseases other than glaucomatous optic nerve damage; and systemic diseases that may affect visual function test results. The eye with less severe disease was selected in subjects with both eyes meeting the inclusion criteria. If both eyes had the same severity, one eye was randomized for the study.

All the subjects underwent a comprehensive ophthalmic examination, including subjective refraction, slit lamp bio microscopy, an IOP examination via Goldmann applanation tonometry, gonioscopy, central corneal thickness (CCT) measurement, and axial length measurement.

Frequency domain optical coherence tomography (FD-OCT, RTVue100, Optovue, Fremont, CA, USA) scans were performed on nondilated pupils with a scanning wavelength of 840 ± 10 nm and a speed of 26,000 A-scans per second. The optic nerve head (ONH) model was used. Three scans of each eye were performed, and only clear, non-reflective images with a signal strength indicator (SSI) of ≥40 were stored. The average RNFL thickness (average RNFL) was recorded.

### 2.2. Visual Field Examination

Humphrey perimetry (Humphrey Field Analyzer model 750i, Carl Zeiss Meditec, Inc., Dublin, CA, USA) was performed, and the Swedish Interactive Threshold Algorithm standard (SITA) 24-2 FAST procedure was used. The test process took about 4–5 min. Each subject underwent at least two consecutive reliable VF examinations. Reliable VF output was defined as a fixation loss rate of <20%, a false-positive rate of <20%, and a false-negative error rate of <30%. To avoid the study curve effect, the results from the second reliable VF examination were selected, and the pattern standard deviation (PSD) and mean deviation (MD) values were recorded.

Visual field damage (positive result) was defined as follows: glaucoma hemifield test (GHT) resulting in “outside normal limits”; in the model deviation probability plot, at least three nonmarginal cluster points in the semi-view field (*p* < 5%), in which at least one point was *p* < 1%; and MD and PSD with *p* < 0.05. Normal VF (negative result) was defined as a GHT resulting in “inside normal limits” and the probability of MD and PSD being within 95% of the normal range.

### 2.3. PVEP Test

PVEP tests were performed (Roland Consult RETIport system, Wiesbaden, Germany, Version 6.11.0.32) by following the International Society for Clinical Electrophysiology of Vision (ISCEV) standard [6]. Reversal black-white checkerboard stimuli with large 1° (60 min) and small 0.25° (15 min) checks were used; the analysis time was 300 ms with an average of 100 sweeps; the filter setting range was 1–100 Hz; and the presentation rate was two reversals/s. The stimuli subtended a 15° visual angle at a test distance of 1 m. The mean luminance was 50 cd/m^2^, and the contrast was 100%. A classical CRT stimulator was used to avoid luminance changes. There was a red fixation cross subtending a visual angle of approximately 0.5° positioned at the center of the field. Before the test, visual acuity was corrected at a viewing distance with one eye covered, and gold cup electrodes comprised a single electrophysiological channel with the following placement: active electrode at O_Z_ (occipital; the subscript _Z_ indicates a middle position); reference electrode at F_Z_ (frontal); and the ground at the left earlobe. The impedance setting was ≤5 k-Ohm. During the recording time, participants were asked to focus on the fixation cross, and at least 80 reversals were recorded. Each eye was recorded three times, and the waveform with the most repeats was selected as the resulting wave. A single test protocol and waveforms analysis took approximately 3 min. Noise response was assessed while the subjects were focused on the unmodulated screen with the same luminance as the stimulus screen. The PVEP signal-to-noise ratio (SNR) was defined as the P100 amplitude to the noise ratio. VEP signals with an SNR of greater than two were included for analysis. In our study, P100 amplitudes and latencies of 1° checks and 0.25° checks were analyzed.

The qualitative results of the P100 amplitude and P100 latency are bounded by a cutoff value set by the instrument: a P100 latency greater than 109 ms was more prolonged than normal; that is, the result was positive. A P100 amplitude of less than 7.7 μV was smaller than normal, and the result was positive.

### 2.4. icVEP Examination

A Neucodia visual electrophysiological device (MKWH-BMD, Huzhou Medconova Medical Technology Co. Ltd., Huzhou, China) was applied with a 24 × 24 array isolated check stimulus signal, and a 10 Hz sinusoidal temporal signal with a 15% positive contrast (the modulation depth was 7.5%; the brightness offset was 7.5%) was chosen for the stimulation. The test field was a 10° visual angle with a 2 × 2 array with a red cross in the center of the screen as the fixation target, and the viewing distance was 55 cm. The framerate was 60 Hz, the luminance was 51 cd/m^2^, and the total number of cycles was 20. A filter with a bandwidth between 1 and 40 Hz was used.

Best visual acuity was also corrected at viewing distance (55 cm) with one eye covered, and gold cup electrodes comprised a single electrophysiological channel. The active electrode was at O_Z_ (occipital), the reference electrode was at C_Z_ (vertex), and the ground was at P_Z_ (parietal). The subjects were asked to focus on the fixed target in the center of the screen during each 2-s-long stimulation. The electroencephalography (EEG) signals in each run were recorded and converted into fundamental frequency components (FFCs) using a discrete Fourier transform. If there was a loss in fixation, significant noise, or other interference in one run, the system would automatically discard the invalid signal and enter the next run. If eight runs were completed and one of the FFCs was out of bounds relative to the other seven signals, the system promoted the “outlier” and automatically discarded the signal. It then started the next run until eight valid FFCs were recorded. The whole process was completed in approximately 2 min. The Neucodia device calculated the mean FFC and the radius of the 95% confidence circle. The icVEP SNR was defined as the ratio of the mean amplitude of the FFC to the radius of the 95% confidence circle, which was the observation index of the icVEP. Three icVEP SNRs were recorded for each eye, and the mean value was calculated.

The threshold value set by the icVEP instrument’s database was used for the qualitative results: icVEP SNR > 1 was a negative result (normal), while icVEP SNR ≤ 1 was a positive result (abnormal).

Each subject was asked to rest for at least 20 min between the VF, PVEP, and icVEP tests to avoid the effects of fatigue.

### 2.5. Statistical Analysis

The general characteristics of the subjects were assessed using SPSS (version 20.0). The Shapiro-Wilk test was used to assess the normality of the continuous variables in each group. Continuous variables were expressed as the means ± standard deviations. Student’s *t*-test, the Mann-Whitney U test, and the chi-square test were used for comparisons between the groups. The McNemar test was used to compare the consistency of the two tests. Receiver operating characteristic (ROC) curve analysis was performed with the DeLong test. The areas under the ROC curve (AUC), the integrated discrimination index (IDI), and the net reclassification index (NRI) were evaluated and compared. When two parameters are compared, an IDI or NRI value of <0 means that the former is worse than the latter, and vice versa. The clinical benefit of different tests in decision-making was evaluated using decision curve analysis (DCA). The AUC, NRI, and IDI were calculated, and DCA was performed using SAS (version 9.0). The ROC curves were determined using MedCalc (version 15.8). The statistical significance level was set as * *p* < 0.05.

## 3. Results

A total of 68 subjects were enrolled, including 33 POAG patients (aged 30–75 years old) and 35 controls (aged 36–75 years old). As Table 1 shows, there were no significant differences in age, sex, axial length, CCT, IOP, or spherical equivalents between the two groups, and the average RNFL of the POAG group was significantly thinner than that of the control group (* *p* < 0.05).

All the parameters of the icVEP, PVEP, and VF tests in the POAG and control groups are summarized in Table 2. In the POAG group, the SNR of icVEP, the P100 amplitude (1° and 0.25° checks) of PVEP, and the MD of VF were significantly smaller, while the P100 latency (0.25° checks) of the PVEP and PSD of the VF were significantly larger than that of the control group ((* *p* < 0.05)). The P100 latency (1° checks) of the PVEP showed no significant difference between the groups (*p* = 0.246).

**Figure 1:**
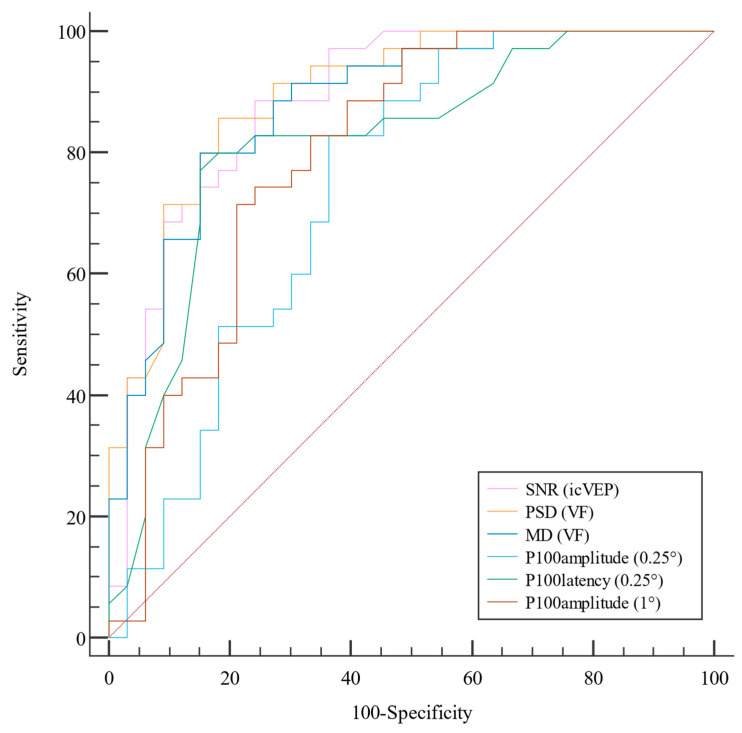
The ROC curves of icVEP, PVEP, and VF. The AUC of PSD was the largest (0.895, 95% CI: 0.821–0.97), followed by those of icVEP SNR (0.887, 95% CI: 0.807–0.967); MD (0.877, 95% CI: 0.796–0.959); P100 latency 0.25° check (0.815, 95% CI: 0.709–0.921); P100 amplitude 1° check (0.798, 95% CI: 0.688–0.908); P100 amplitude 0.25° check (0.742, 95% CI: 0.62–0.864); and last, P100 latency 1° check (0.582, 95% CI: 0.431–0.732. The latter is not listed in the curve because it was much worse than the other parameters).

As Figure 1 and Table 3 show, the AUC of PSD was the largest (0.895), followed by that of the icVEP SNR (0.887), the MD (0.877), the P100 latency of 0.25° checks (0.815), the P100 amplitude of 1° checks (0.798), the P100 amplitude of 0.25° checks (0.742), and the P100 latency of 1° checks (0.582). The specificity of the SNR (88.57%) was the highest of all the parameters, while the MD and P100 latency (0.25° checks) displayed higher sensitivities (both 84.85%) than the other parameters. The PSD had the highest diagnostic accuracy (0.838), icVEP SNR had the highest positive predictive value (PPV, 86.2%), and MD had the highest negative predictive value (NPV, 84.8%).

The discriminative capability of the icVEP, PVEP, and VF tests are analyzed in Table 4. We compared the AUC, IDI, and NRI of the icVEP SNR to those of VF and PVEP assessment outcomes. We found that in the comparisons of PSD vs. icVEP SNR, MD vs. icVEP SNR, and P100 latency (0.25°) vs. icVEP SNR, the ∆AUC, IDI, and NRI showed no significant difference (*p* > 0.05). However, in the comparison of P100 amplitude (1°) with icVEP SNR, the IDI value was −0.197 (* *p* = 0.005). Additionally, the AUC and IDI in the comparison of P100 amplitude (0.25°) with icVEP SNR (AUC difference value = −0.145, *p* = 0.015; IDI = −0.277, * *p* < 0.001) and the comparison of P100 latency (1°) with icVEP SNR (AUC difference value = −0.305, * *p* ≤ 0.001; IDI = −0.368, * *p* < 0.001) showed significant differences. The results showed that icVEP SNR has the same diagnostic value as the PSD and MD of VF and the P100 latency (0.25°) of PVEP, and it has a better diagnostic value than other parameters of PVEP.

In the qualitative comparison, as shown in Table 5, the sensitivity and specificity of the icVEP, VF, and P100 latency (0.25°) of PVEP were close to each other. The sensitivity of P100 latency (0.25°) was the highest (78.79%), and the specificity of the icVEP was the highest (82.86%). The other variables of PVEP have good specificity but poor sensitivity.

P100 latency (0.25°) offers the best diagnostic ability among the PVEP parameters and, therefore, was selected for consistency analysis with the qualitative results of icVEP and VF. As shown in Table 6, the paired chi-square test showed that there was no significant difference between the three examinations (McNemar *p* > 0.05).

**Figure 2:**
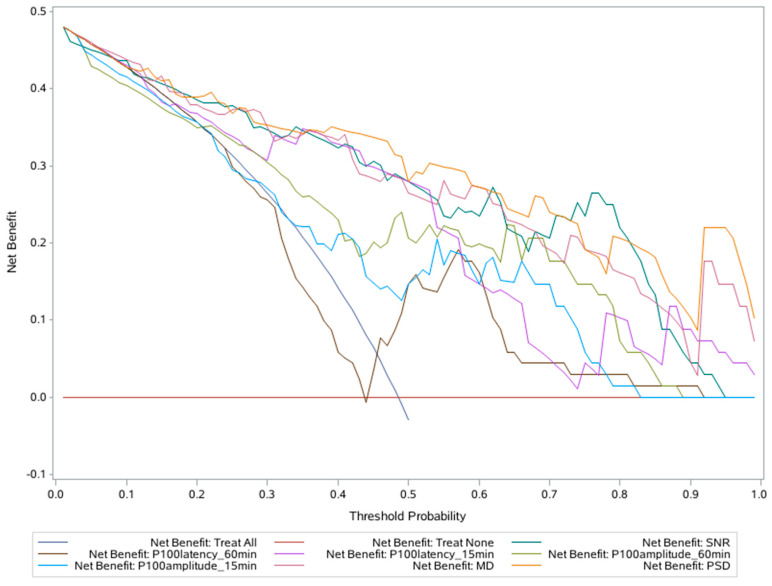
DCA of icVEP, PVEP, and VF.

A DCA was performed to assess the clinical utility of the above three tests. As the net benefit (NB) curves (Figure 2) show, two lines represent two extremes: the diagonal line in dark blue represents all subjects with POAG (treat all), while the horizontal red line represents none of the subjects with POAG (treat none: non-POAG). A parameter was deemed the best indicator if it showed a maximum NB (ordinate) value at any given threshold (abscissa). Therefore, the three curves of PSD, SNR, and MD were the farthest from the two extreme curves, indicating that the VF and icVEP tests offer more clinical benefit value than the PVEP test.

## 4. Discussion

In this study, the role of icVEP in the diagnosis of early and moderate POAG was evaluated by comparison with VF and PVEP, and glaucomatous optic neuropathy was used as the criterion for POAG.

POAG is characterized by progressive damage to RGCs and their axons, resulting in visual function damage [23]. VF assessments can provide topographic information and are widely used for the detection and follow-up of glaucomatous visual impairment [24]. In our study, the sensitivities (PSD: 81.82%, MD: 84.85%) and specificities (PSD: 85.71%, MD: 80%) of VF tests for distinguishing POAG patients from non-POAG patients were close to the results of several previous studies [25,26].

VEPs are visually evoked electrophysiological signals obtained from EEG activity in the visual cortex that can reflect the integrity of the entire visual pathway from the retina to the occipital cortex [6]. VEP tests have been used in glaucoma research and have been shown to offer potential diagnostic value [3,27,28,29,30]. Increased P100 latency and decreased P100 amplitude in PVEPs have both been reported in glaucoma subjects in previous studies [3,4,31,32,33]. In our study, the P100 latency and P100 amplitude parameters, which were demonstrated to be more sensitive in previous studies [3], were selected for analysis. Among the parameters assessed by PVEP, a P100 latency of 0.25° checks had the largest AUC (0.815) in distinguishing POAG from non-POAG in our results.

The results of this study showed that the POAG group had a significantly smaller icVEP SNR than the control group (* *p* < 0.05, Table 2) and that the icVEP SNR had good diagnostic capability for POAG (Table 3), a result which was also indicated in previous studies [7,8,9,10,11,12,13]. However, the AUC (0.887), sensitivity (75.76%), and specificity (88.57%) of the icVEP SNR to discriminate POAG patients from controls in our study were slightly lower than those of Kolomeyer et al. (the AUC, sensitivity, and specificity of the icVEP SNR were 0.94, 82%, and 100%, respectively) [12]. In addition, Fan et al. [11] showed that, with an icVEP SNR≤1 as the positive cutoff value, the sensitivity to diagnose POAG was 62.2%, which was lower than the results of this study (72.73%). The sensitivities and specificities of the icVEP SNR for the diagnosis of POAG could not be compared between various studies because different research equipment, testing environments, and population enrollment would have influenced the results.

In our qualitative comparison, the sensitivity and specificity of the icVEP, VF, and P100 latency (0.25° checks) of PVEP were similar. The sensitivities were as follow: icVEP, 72.73%; VF, 75.76%; and PVEP, 78.79%. The specificities were as follow: icVEP, 82.86%; VF, 80%; and PVEP, 80% (Table 5). No significant difference was found in our consistency analysis of the qualitative comparison between the three (McNemar *p* > 0.05). Chen et al. [9] made a qualitative comparison between icVEP and SAP and found that icVEP and SAP had similar sensitivity and specificity regarding diagnostic ability in POAG; however, the sensitivities in their study (SAP, 59.2%; icVEP, 53.1%) were slightly lower than those of ours. This outcome may be because of the different inclusion criteria of POAG patients and inspection equipment. Chen et al. used MKWH-AMD equipment in their study, and glaucomatous optic nerve damage combined with the Moorfields Regression Analysis (MRA) classification of HRT was used as the standard. In the present study, a new generation of MKWH-BMD equipment was used, and glaucomatous optic nerve damage combined with RNFL defects was taken as the standard. In the study by Parisi et al. [3], the P100 latency and amplitude of PVEP in POAG patients were found to be significantly correlated with the MD of SAP. However, there have been no reports on qualitative comparisons of PVEPs and VFs and comparisons of icVEP and PVEPs in glaucoma populations thus far. For the first time, this study demonstrated the consistency of icVEP, PVEP, and VF in the detection of glaucoma, suggesting that all three of these functional test methods can detect optic nerve damage in glaucoma. However, the clinical application of icVEP in glaucoma needs more and further studies for confirmation.

Table 3 shows that the SNR of the icVEP test has a similar capability in discriminating POAG patients from controls as the PSD and MD of the VF test and the P100 latency (0.25° checks) of the PVEP assessment. A qualitative comparison between icVEP and VF by Chen et al. [9] showed a similar result, in which icVEP and VF had similar sensitivity and specificity in the diagnosis of POAG. Parisi et al. [3] found that the P100 latency and amplitude of PVEP assessments were significantly correlated with the MD of VF in POAG patients. However, no comparative study on icVEP and PVEP assessments in the glaucoma populations has been reported. Theoretically, a detection method with better diagnostic ability provides better guidance for clinical decision-making. However, statistical methods such as ROC curve analysis are insufficient for assessing whether a test has a clinical benefit value [34]. A DCA was performed to further assess the clinical benefits of the icVEP, PVEP, and VF parameters. As Figure 2 shows, the PSD and MD of VF and the SNR of icVEP demonstrated a higher NB under a given threshold probability, indicating that patients could benefit more, clinically, from VF and icVEP tests.

The advantages of icVEP are that it does not require human–machine conversations, and the fixation time is short, making it a possible, important alternative diagnostic method for POAG, especially for populations who have difficulty cooperating with VF examination, such as those who cannot understand the VF testing process or those who have poor hand–eye coordination or upper limb disability. Our previous study [35] showed that icVEP offers good diagnostic capabilities for POAG in a high-myopia population. Thus, it may be suitable for evaluating special populations. However, as a kind of visual electrophysiological test, icVEP results may also be affected by environmental factors, such as the lighting of the examination room, ambient noise, other electrical operations, etc., and the diagnosis of POAG requires the exclusion of other diseases with visual-pathway abnormalities. In addition, the icVEP test requires BCVA ≥ 20/40, which limits its clinical use in opaque interstitial populations, such as those with cataracts. The disadvantage of icVEP is that it has only one index and is not suitable for quantitative evaluation and follow-up. Our previous study found that the sensitivity of icVEP is low in early glaucoma populations who present with peripheral visual field defects [35]. Therefore, icVEP cannot replace the visual field test. In addition, the disadvantage of this study is that the sample size included was relatively small, and larger samples are needed in the future to further confirm the reliability of the current results.

## 5. Conclusions

According to our study, icVEP, VF, and PVEP tests have similar abilities to distinguish POAG and non-POAG patients. VF and icVEP tests could offer patients more clinical benefits. Thus, icVEP can be used as an effective supplement to VF examination because of its similar diagnostic capability and clinical benefits in POAG patients.

## Figures and Tables

**Table 1:** Sociodemographic and clinical characteristics of the participants.

Variables	POAG N = 33	Control N = 35	*p*
Age, year	56.39 ± 11.3	56.11 ± 8.56	0.792 ^c^
Sex (male/female)	18/15	13/22	0.15 ^b^
Axial length, mm	23.92 ± 1.3	23.32 ± 1.3	0.059 ^a^
CCT, μm	547.91 ± 28.31	539.31 ± 35.53	0.39 ^a^
IOP, mmHg	14.67 ± 2.15	15.11 ± 2.38	0.343 ^c^
Spherical equivalent, D	−1.46 ± 2.7	−0.5 ± 2.4	0.121 ^c^
Average RNFL, μm	85.22 ± 11.55	110.4 ± 10.96	<0.001 ^a^

The data are expressed as the means ± SDs; ^a^: independent samples *t*-test; ^b^: chi-square test; ^c^: Mann–Whitney U test; POAG: primary open-angle glaucoma; CCT: central corneal thickness; IOP: intraocular pressure; RNFL: thickness of retinal nerve fiber layer.

**Table 2:** Measurements of icVEP, PVEP, and VF parameters in the POAG and control groups.

Variables	POAG N = 33	Control N = 35	*p*
icVEP			
SNR	0.87 ± 0.32	1.57 ± 0.53	<0.001 ^b^
PVEP			
P100 latency (1°), ms	106.6 ± 9.28	103.54 ± 2.9	0.246 ^a^
P100 latency (0.25°), ms	116.56 ± 10.07	106.96 ± 5.69	<0.001 ^b^
P100 amplitude (1°), μv	8.62 ± 5.44	13.39 ± 4.5	<0.001 ^b^
P100 amplitude (0.25°), μv	10.18 ± 6.72	14.5 ± 4.88	0.001 ^b^
VF			
MD, dB	−5.55 ± 3.21	−1.81 ± 1.5	<0.001 ^b^
PSD, dB	5.32 ± 3.04	1.86 ± 0.93	<0.001 ^b^

The data are expressed as the means ± SDs; ^a^: independent samples *t*-test ^b^: Mann–Whitney U test; icVEP: isolated-check visual evoked potential; PVEP: pattern visual evoked potential; SNR: signal-to-noise ratio; VF: visual field; MD: mean deviation; PSD: pattern standard deviation.

**Table 3:** ROC of the parameters of the icVEP, PVEP, and VF tests.

Parameters	AUC	95% CI	Best Threshold	Sensitivity, %	Specificity, %	Accuracy	PPV, %	NPV, %
PSD, db (VF)	0.895	(0.821, 0.97)	2.725	81.82	85.71	0.838	84.4	83.3
SNR (icVEP)	0.887	(0.807, 0.967)	0.99	75.76	88.57	0.823	86.2	79.5
MD, db (VF)	0.877	(0.796, 0.959)	−2.81	84.85	80	0.824	80	84.8
P100 latency (0.25°), ms (PVEP)	0.815	(0.709, 0.921)	108.5	84.85	77.14	0.809	77.8	84.4
P100 amplitude (1°), μv (PVEP)	0.798	(0.688, 0.908)	10.75	78.79	71.43	0.778	72.2	78.1
P100 amplitude (0.25°), μv (PVEP)	0.742	(0.620, 0.864)	10.57	63.64	82.86	0.722	77.8	70.7
P100 latency (1°), ms (PVEP)	0.582	(0.431, 0.732)	109	39.39	85.71	0.485	72.2	60

AUC: area under the curve; CI: confidence interval; PPV: positive predictive value; NPV: negative predictive value. PSD: pattern standard deviation; SNR: signal-to-noise ratio; MD: mean deviation.

**Table 4:** AUC, NRI, and IDI of the icVEP, PVEP, and VF tests.

Parameter of Comparison		Value	95%CI	*p*
VF PSD vs. icVEP SNR	∆AUC	0.009	(−0.101, 0.118)	0.877
	IDI	0.066	(−0.102, 0.235)	0.439
	NRI	−0.007	(−0.271, 0.257)	0.96
VF MD vs. icVEP SNR	∆AUC	−0.009	(−0.126, 0.108)	0.879
	IDI	0.013	(−0.15, 0.176)	0.874
	NRI	−0.036	(−0.319, 0.248)	0.809
PVEP P100 latency (0.25°) vs. ic VEP SNR	∆AUC	−0.072	(−0.211, 0.067)	0.311
	IDI	−0.105	(−0.268, 0.058)	0.208
	NRI	−0.005	(−0.286, 0.275)	0.971
PVEP P100 amplitude (1°) vs. icVEP SNR	∆AUC	−0.088	(−0.209, 0.0324)	0.152
	IDI	−0.197	(−0.336, 0.059)	0.005
	NRI	−0.152	(−0.422, 0.119)	0.28
PVEP P100 amplitude (0.25°) vs. icVEP SNR	∆AUC	−0.145	(−0.262, 0.028)	0.015
	IDI	−0.277	(−0.398, 0.156)	<0.001
	NRI	−0.268	(−0.533, 0.002)	0.056
PVEP P100 latency (1°) vs. icVEP SNR	∆AUC	−0.305	(−0.47, 0.139)	<0.001
	IDI	−0.368	(−0.494, 0.242)	<0.001
	NRI	−0.278	(−0.563, 0.007)	0.081

∆AUC: difference in area under the curve; IDI: integrated discrimination index; NRI: net reclassification index; VF: visual field; PSD: pattern standard deviation; icVEP: isolated-check visual evoked potential; SNR: signal-to-noise ratio; MD: mean deviation; PVEP: pattern visual evoked potential.

**Table 5:** Qualitative comparison of icVEP, VF, and PVEP parameters.

Parameters	Sensitivity (%)	Specificity (%)
icVEP	72.73	82.86
VF	75.76	80
P100 latency (0.25°)	78.79	80
P100 amplitude (0.25°)	36.36	97.14
P100 latency (1°)	39.39	100
P100 amplitude (0.25°)	51.52	97.14

icVEP: isolated-check visual evoked potential; VF: visual field.

**Table 6:** Consistency comparison of icVEP, VF, and PVEP (P100 latency of 0.25°).

		VF		McNemar p	PVEP (P100 Latency of 0.25°)		McNemar p
		+	-		+	-	
icVEP	+	18	12	0.845	21	9	0.523
	-	14	24		13	25	
PVEP(P100 latency, 0.25°)	+	21	12	0.839	NA	NA	NA
	-	11	23		NA	NA	

“+” means positive result; “-” means negative result. VF: visual field; PVEP: pattern visual evoked potential; icVEP: isolated-check visual evoked potential; NA: not applicable.

## Data Availability

Data are available upon request.
